# Research on the Acoustic Scattering Characteristics of Underwater Corner Reflector Linear Arrays

**DOI:** 10.3390/s25072129

**Published:** 2025-03-27

**Authors:** Dawei Xiao, Jingzhuo Zhang, Zichao Chu, Yi Luo

**Affiliations:** 1 Naval University of Engineering, Wuhan 430033, China; david_engineer@126.com (D.X.); luomx@163.com (Y.L.); 2 Naval Aviation University, Yantai 264001, China

**Keywords:** corner reflector linear array, acoustic scattering characteristics, target strength, deployment optimization, improvement of reflection blind zones

## Abstract

This manuscript aims to optimize the acoustic scattering characteristics of underwater corner reflector linear arrays through simulation analysis and experimental validation, thereby enhancing their application efficiency in underwater acoustic countermeasures, particularly in terms of increasing acoustic echo intensity and reducing reflection blind spots. The acoustic scattering characteristics of submerged corner reflectors were meticulously simulated using the finite element method–boundary element method coupling technique, and the simulation results were rigorously verified through tank experiments. The study focused on the impact of the number of corner reflectors and their deployment angles on acoustic echo characteristics. Simulation and experimental results revealed that increasing the number of corner reflectors significantly enhances the overall target strength, with a dual corner reflector array achieving an approximately 5 decibels higher target strength than a single corner reflector. Moreover, the interaction of scattered acoustic waves among corner reflectors in the linear array generates noticeable fluctuations in the target strength curve, with these fluctuations increasing in frequency as the number of corner reflectors rises. By judiciously adjusting the deployment angles of the corner reflectors to achieve complementarity between strong and weak reflection angles, the issue of reduced target strength near 5° and 85° can be effectively mitigated, thereby significantly reducing reflection blind spots.

## 1. Introduction

**Figure 1:**
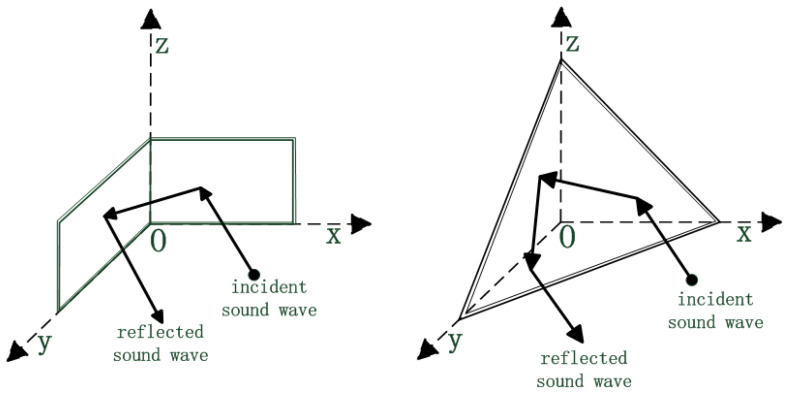
Schematic diagram of working principle of corner reflectors. (Standard dihedral rectangular and trihedral triangular corner reflectors).

Corner reflectors have achieved relatively complete development in the field of radar countermeasures due to their excellent electromagnetic wave backscattering characteristics. Radar corner reflectors, characterized by low cost and easy implementation, are widely used in passive jamming. Their purpose is to form a large radar cross-section [1,2,3]. Their typical structure consists of mutually orthogonal metal reflecting surfaces (as shown in Figure 1). According to their geometric configuration, they can be classified into two types: typical trihedral corner reflectors and dihedral corner reflectors, as well as combined multi-face corner reflectors, which can significantly increase the scattering intensity and the effective scattering angle [4,5,6,7,8,9]. Their core mechanism stems from the “cavity resonance effect”: when the incident electromagnetic wave satisfies the phase-matching condition of λ ≈ 2L/n (where λ is the wavelength, L is the side length of the cavity, and n is an integer) with the cavity structure, it will excite the coherent superposition of surface currents, and through three specular reflections, a stable echo-enhancement mechanism is formed [10,11]. Since the reflection range of a single corner reflector has certain limitations, and under the condition that other parameters are the same, the target strength of a single corner reflector is relatively low. By reasonably arranging corner reflectors to form false targets, we can accurately simulate the radar scattering characteristics of our military forces (such as aircraft), deceive enemy-guided weapons to attack these false targets, and protect our military equipment [12].

Corner reflectors have been widely used in countering electromagnetic-guided weapons, radars, etc. However, their applications in the field of underwater countermeasures are still relatively scarce. The domestic research on underwater corner reflectors is described below:

Chen Wenjian [13] studied the acoustic reflection of metal plates and proposed the elastic skip method for acoustic wave beams. This method takes into account the multiple scattering of acoustic waves within the “concave”-shaped structure of the corner reflector, making up for the defects of other calculation methods that only calculate single-acoustic scattering, and improving the calculation accuracy.

Liang Jingjing [14] corrected the acoustic beam skip method. In order to achieve a faster calculation speed, the elastic skip method for acoustic wave beams with rapid estimation was used, simplifying the calculation steps and enhancing the calculation efficiency. However, the elastic skip method for acoustic wave beams regards the target as a non-elastic body and does not consider the elastic effect of the structure itself. The analysis conditions are relatively idealized, and it can be used as an estimation method under high-frequency conditions [15].

Reference [16]’s contribution is to present an isogeometric, frequency-stable algorithm for the solution of acoustic obstacle scattering problems with essential linear complexity in terms of the number of boundary elements and potential evaluation points. By the Dirichlet-to-Neumann (DtN) operator, an exact transparent boundary condition is introduced and the model is formulated as a boundary value problem of acoustic–elastic interaction. Based on a duality argument technique, an a posteriori error estimate is derived for the finite element method with the truncated DtN boundary operator. The a posteriori error estimate consists of the finite element approximation error and the truncation error of the DtN boundary operator, where the latter decays exponentially with respect to the truncation parameter. An adaptive finite element algorithm is proposed for solving the acoustic–elastic interaction problem, where the truncation parameter is determined through the truncation error and the mesh elements for local refinements are chosen through the finite element discretization error. Numerical experiments are presented to demonstrate the effectiveness of the proposed method [17].

The flat-plate materials of radar corner reflectors generally use thin metal sheets. However, thin metal sheets have good sound-transmission properties, poor sound-reflection performance, a low echo target strength underwater, and obvious elastic characteristics [18]. Their direct application in the underwater acoustic field is not satisfactory. A technical method to enhance the sound-reflection ability of corner reflectors and reduce the energy loss of acoustic wave transmission will make the difference in characteristic impedance between the material of the reflecting surface and the water as large as possible and maximize the reflection coefficient of the reflecting surface, and using a low-impedance material as an interlayer can effectively improve the sound-reflection intensity of underwater corner reflectors [19,20].

In the actual processing of corner reflectors, errors are inevitable, resulting in the flat plates forming the corner reflectors not being completely perpendicular. Taotao Xie carried out simulation calculations on non-orthogonal corner reflectors of flat plates and compared them with orthogonal corner reflectors. The results show that the sound-reflection ability of orthogonal corner reflectors is stronger than that of non-orthogonal corner reflectors, and when the processing error of the flat plates of non-orthogonal corner reflectors is controlled within 2°, it will not have a significant impact on the acoustic scattering ability of the corner reflectors [21].

**Figure 2:**
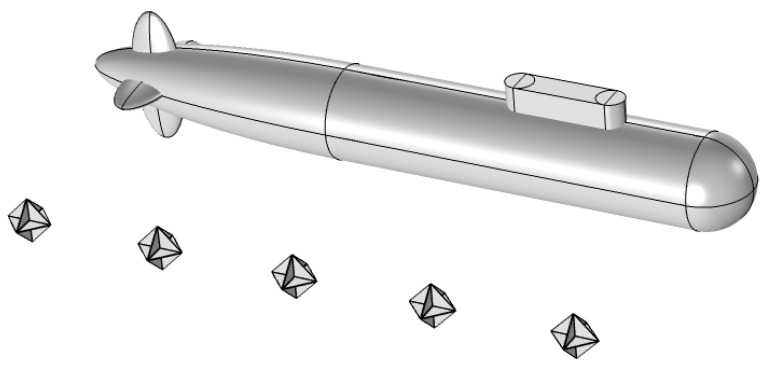
Schematic diagram of corner reflector linear array simulating submarine acoustic scattering. (Using corner reflector array to simulate multiple bright spots of submarine echoes).

The aforementioned research primarily focuses on enhancing the acoustic scattering characteristics of individual corner reflectors. However, in underwater countermeasures, it is typically necessary to simulate the acoustic scattering characteristics of naval vessels to construct decoy targets. These decoys are designed to deceive acoustic homing systems of underwater guided weapons, causing them to track and engage with the false targets instead. Given the considerable length of naval vessels, using a single corner reflector can only simulate point reflection characteristics and is not sufficiently accurate for replicating the volume scattering features of a vessel. Since underwater guided weapons generally identify targets by the scale of multiple echo highlights from a certain direction, this manuscript adopts for the first time the method of combining multiple corner reflectors in series to form a linear array to simulate the scale reflection characteristics of ships. The principle is shown in Figure 2.

This manuscript concentrates on enhancing the acoustic scattering characteristics of underwater corner reflector linear arrays, which presents a more complex and challenging domain compared to the study of individual corner reflectors. Although many studies have simulated the acoustic scattering properties of single corner reflectors and found them to exhibit regularity under certain angles of incidence, the overall acoustic scattering characteristics become significantly more complex when dealing with linear arrays composed of multiple corner reflectors due to their intricate geometric structure and the inevitable interactions among the reflectors.

Particularly in underwater environments, the mutual influence between adjacent reflectors in a corner reflector linear array is especially significant, playing a decisive role in the acoustic scattering properties. To tackle this issue, this study innovatively proposes adjusting the deployment distance and relative angles of the corner reflectors to conduct acoustic scattering characteristic simulations for both two-element and three-element corner reflector linear arrays. By investigating the acoustic scattering properties of single-layer metal corner reflector arrays and integrating acoustic scattering enhancement methods from the literature [19,20,21], we perform pool experiments with air-cavity corner reflectors known for their superior acoustic performance. This approach provides a theoretical basis for more accurately simulating the acoustic characteristics of large targets such as naval vessels and constructing effective underwater decoy targets.

Ultimately, based on the simulation outcomes, this study will propose a set of design and optimization methods for underwater corner reflector linear arrays. This not only enriches the theoretical foundation of acoustic scattering characteristic research in the field of corner reflector linear arrays, but also offers a novel technical approach for the construction of decoy targets in underwater countermeasures, holding significant practical value and extensive development prospects.

## 2. Calculation Methods for the Acoustic Scattering of Underwater Targets

The finite element method–Boundary Element Method (FEM—BEM) stands as a highly potent computational approach for the acoustic scattering of non-rigid bodies at present. It has the capacity to solve the problem of the vibration-coupled acoustic radiation of underwater targets within an infinite-domain fluid field [22]. The core idea involves leveraging the finite element method (FEM) to conduct an in-depth analysis of the vibration response of the target structure and applying the boundary element method (BEM) to meticulously analyze the vibro-acoustics of the target. By integrating these two methods to constitute the finite element–boundary element coupling method (FEM—BEM), the acoustic–vibration coupling problem of the target can be comprehensively analyzed [23,24]. This methodology not only ensures computational accuracy, but also remarkably boosts computational speed. Consequently, utilizing the FEM-BEM to compute the acoustic scattering problem of elastic targets in an infinite fluid domain presents substantial advantages.

### 2.1. The Acoustic Reflection Properties of Metal Thin Plates

**Figure 3:**
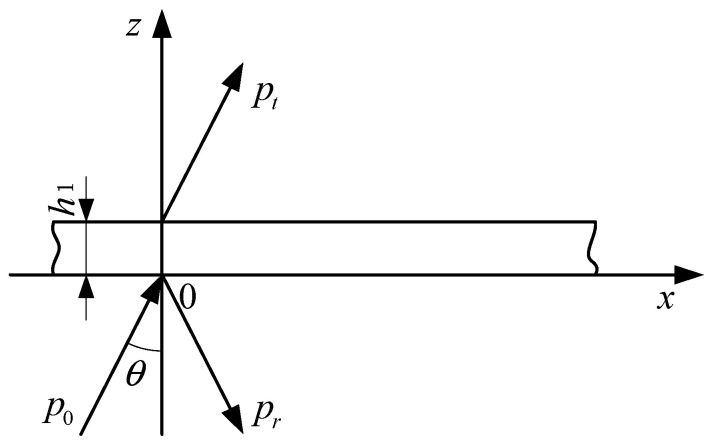
Schematic diagram of propagation of sound wave through thin plate.

Figure 3 illustrates the schematic diagram of sound wave propagation through a thin plate, where *h*_1_ represents the thickness of the thin plate, *P*_0_ and *P*_t_ denote the incident wave and transmitted wave, respectively, *P*_r_ stands for the reflected wave, and *θ* is the angle between the incident wave and the positive direction of the *z*-axis.

For slabs whose thickness is much smaller than the wavelength of sound waves in the material, they can be treated as thin plates when calculating their reflection coefficients.

(1) $$A=\frac{ZZ_{np}-4{Z_{0}}^{2}}{\left(Z+2Z_{0})(Z_{np}+2Z_{0}\right)}$$

In the formula, *A* represents the reflection coefficient, *Z* is the acoustic impedance of the thin plate’s flexural vibration, *Z_np_* denotes the acoustic impedance for the symmetric vibration of the thin plate, and *Z*_0_ = *ρc*/cos(*θ*) is the normal acoustic impedance, where *ρ* is the density of water and *c* is the speed of sound in water.

In practice, for any metallic thin plate, *Z_np_* is much greater than *Z*_0_ across nearly all ranges of incidence angles. Therefore, an approximation that disregards shear waves can be used to calculate the reflection coefficient.

(2) $$A=\frac{Z}{Z+2Z_{0}}$$

$Z=-i\omega \rho_{M}h(1-\frac{{c_{H}}^{4}}{c^{4}}\sin^{4}\theta )$; therefore,

(3) $$\left|A\right|=\frac{1}{\sqrt{1+{\left\{\frac{2\rho c}{\omega \rho_{M}h\cos\theta \left[1-{\left(c_{H}/c\right)}^{4}\sin^{4}\theta \right]}\right\}}^{2}}}$$

where ${c_{H}}^{4}=\frac{\omega^{2}Eh^{2}}{12(1-\sigma^{2})\rho_{M}}$, *ρ_M_* is the material density, *h* is the thickness of the plate, *ω* = 2π*f*, *f* is the frequency of the incident wave, *E* is the elastic modulus, and *σ* is the Poisson’s ratio of the material.

### 2.2. Anti-Acoustic Performance of the Air Layer

**Figure 4:**
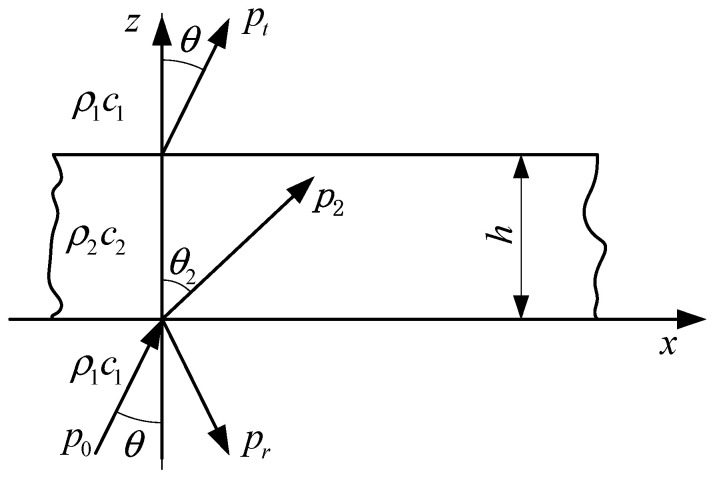
Schematic diagram of propagation of sound wave through air layer.

Figure 4 is a schematic diagram of the propagation of sound waves through an air layer in water. Here, *h* is the thickness of the air layer, *P*_0_ and *P_t_* are the incident and transmitted waves, respectively, *P_r_* is the reflected wave, *P*_2_ is the refracted wave, *θ* is the angle of incidence, *θ*_2_ is the angle of refraction, *ρ*_1_ is the density of water, *c*_1_ is the speed of sound in water, *ρ*_2_ is the density of air, and *c*_2_ is the speed of sound in air. The reflection coefficient is

(4) $$A=\frac{i(1/w-w)\sin P}{2\cos P-i(1/w+w)\sin P}$$

In the formula, *P* = *k*_2_*h*cos*θ*_2_, where *k*_2_ is the wavenumber of the sound wave in air, $w=\frac{\rho_{2}c_{2}\cos\theta }{\rho_{1}c_{1}\cos\theta_{2}}$, and $\cos\theta_{2}=\sqrt{1-\frac{{c_{2}}^{2}}{{c_{1}}^{2}}\sin^{2}\theta }$.

If the angle of incidence of the plane wave does not exceed the critical angle for total internal reflection and *P* is a real number, then

(5) $$\left|A\right|=\frac{\left|(1/w-w)\sin P\right|}{\sqrt{4\cos^{2}P+{\left(1/w+w\right)}^{2}\sin^{2}P}}$$

### 2.3. Calculation Methods for Acoustic Scattering by Underwater Corner Reflectors

An underwater corner reflector is a thin-walled structure immersed in water. The finite element method (FEM) for structures, coupled with the indirect boundary element method (BEM) for fluids, is suitable for solving acoustic scattering problems involving non-enclosed structures or structures with fluid both inside and outside. Therefore, this method is employed to calculate the acoustic scattering of underwater acoustic corner reflectors. Below, the fluid–solid coupling equations of this method are derived.

**Figure 5:**
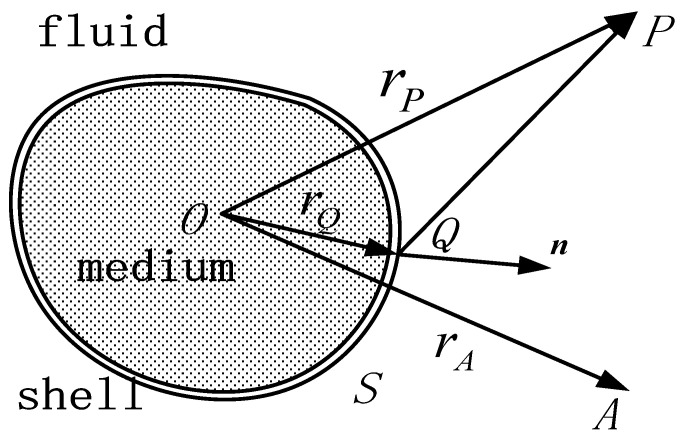
Geometric relationship of IBEM.

Assuming that the object is in an ideal fluid, *Q* is an arbitrary point on the structural surface *S*, ***n*** is the unit normal vector pointing outward at point *Q*, and *P* is an arbitrary point in space, *r_P_* is the distance between point *O* and point *P*, *r_A_* is the distance between point *O* and point *A*. The schematic diagram is shown in Figure 5.

The Helmholtz equation for the single-frequency scattered sound field using the direct boundary element method can be derived from the wave equation.

(6) $$\iint_{S}\left[p(r_{Q})\frac{\partial G(r_{P},r_{Q})}{\partial n}+j\omega \rho v_{n}G(r_{P},r_{Q})\right]dS=\left\{\begin{array}{ll} p(r_{P}) & P\;\mathrm{is}\;\mathrm{outside}\;S.\\ \frac{1}{2}p(r_{P}), & P\;\mathrm{is}\;\mathrm{on}\;S.\\ 0 & P\;\mathrm{is}\;\mathrm{inside}\;S \end{array}\right.$$

In the equation, *p* is the instantaneous sound pressure, $p(r_{Q})$ is the sound pressure at point *Q*, $G(r_{P},r_{Q})=e^{-jk\left|r_{P}-r_{Q}\right|}/4\pi \left|r_{P}-r_{Q}\right|$ is the Green’s function, *k* = *ω*/*c* is the wavenumber, *ω* is the angular frequency, *c* is the speed of sound in the fluid, *ρ* is the fluid density, *v_n_* is the normal vibration velocity at point *Q*, and $p(r_{P})$ is the sound pressure at point *P*.

The indirect boundary element method (BEM) can be derived from the direct BEM. In the indirect BEM, the unknown variables are the difference in sound pressure on both sides of the boundary surface (double-layer potential) and the difference in the gradient of sound pressure (single-layer potential). By applying the Helmholtz integral equation of the direct BEM to both sides of the boundary surface and then subtracting the two equations, the sound pressure at any field point can be obtained.

(7) $$p(r_{P})=\iint_{S}\left[G(r_{P},r_{Q})\sigma (r_{Q_{1}},r_{Q_{2}})-\frac{\partial G(r_{P},r_{Q})}{\partial n_{Q}}\mu (r_{Q_{1}},r_{Q_{2}})\right]dS$$

In the equation, $\sigma (r_{Q_{1}},r_{Q_{2}})$ and $\mu (r_{Q_{1}},r_{Q_{2}})$ represent the difference in the normal pressure gradient and the pressure difference on both sides of the surface, respectively.

(8) $$\begin{array}{l} \sigma (r_{Q_{1}},r_{Q_{2}})=\frac{\partial p(r_{Q_{1}})}{\partial n}-\frac{\partial p(r_{Q_{2}})}{\partial n}\\ \;=-j\rho \omega \left[v(r_{Q_{1}})-v(r_{Q_{2}})\right] \end{array}$$

(9) $$\mu (r_{Q_{1}},r_{Q_{2}})=p(r_{Q_{1}})-p(r_{Q_{2}})$$

Assuming that the boundary surface satisfies the Neumann boundary condition and defining point *P* on the boundary, the relationship between the boundary condition and the unknown variable can be derived as

(10) $$\begin{array}{l} \frac{\partial p(r_{P})}{\partial n_{P}}=\iint_{S}\left[\frac{\partial G(r_{P},r_{Q})}{\partial n_{P}}\sigma (r_{Q_{1}},r_{Q_{2}})-\frac{\partial G(r_{P},r_{Q})}{\partial n_{P}\partial n_{Q}}\mu (r_{Q_{1}},r_{Q_{2}})\right]dS\\ \;=-j\rho \omega v(r_{P}) \end{array}$$

In the equation, $v(r_{P})$ is the vibration velocity at point *P*. By discretizing the boundary surface, the unknown variables on the boundary element model surface can be expressed in terms of the unknown variables of the model nodes and shape functions. Based on the variational principle, the general form can be derived.

(11) $$\left[A\right]\left\{x\right\}=\left\{F_{a}\right\}$$

In the equation, $\left[A\right]$ is a symmetric matrix, $\left\{x\right\}$ represents the unknown variables on the boundary element model surface, that is, $\sigma (r_{Q_{1}},r_{Q_{2}})$ or $\mu (r_{Q_{1}},r_{Q_{2}})$, and $\left\{F_{a}\right\}$ is the acoustic excitation vector function.

For the acoustic scattering problem of an underwater elastic target, assuming that the structural damping is zero, the lossless coupled system equation of the finite element structural model and the indirect boundary element fluid model in the physical coordinate system is

(12) $$\left[\begin{array}{cc} \left[K_{s}\right]-\omega^{2}\left[M_{S}\right] & -\left[R_{sf}^{e}\right]\\ -\rho_{s}\omega^{2}{\left[R_{sf}^{e}\right]}^{T} & \left[A\right] \end{array}\right]\left\{\begin{array}{c} \left\{U^{e}\right\}\\ \left\{\delta p^{e}\right\} \end{array}\right\}=\left\{\begin{array}{c} \left\{F_{s}^{e}\right\}\\ \left\{0\right\} \end{array}\right\}$$

In the equation, $\left[K_{s}\right]$ is the structural stiffness matrix, $\left[M_{S}\right]$ is the structural mass matrix, ${\left[R_{sf}^{e}\right]}^{T}$ is the element fluid–structure coupling matrix, $\rho_{s}$ is the structural density, $\left\{U^{e}\right\}$ is the nodal displacement vector, $\left\{\delta p^{e}\right\}$ is the nodal acoustic pressure variation, and $\left\{F_{s}^{e}\right\}$ is the external force matrix on the structural nodes.

After calculating the unknown variables on the boundary surface, the sound pressure and sound intensity at any field point in space can be solved, thereby obtaining the corresponding target strength values.

(13) $$TS=10\mathrm{lg}{\left|\frac{I_{r}}{I_{i}}\right|}_{r=1}$$

In the equation, ${\left.I_{r}\right|}_{r=1}$ is the scattered sound intensity, calculated based on the spherical wave propagation law at a distance of 1 m from the equivalent acoustic center of the target, and $I_{i}$ is the incident sound intensity.

## 3. Simulation-Based Computation

**Figure 6:**
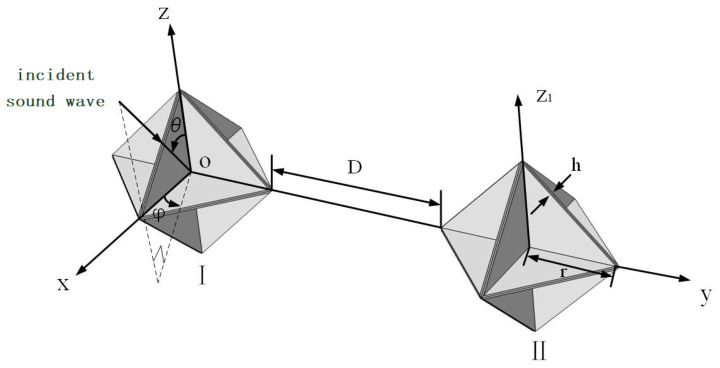
Schematic diagram of two corner reflector arrays.

When acoustic waves impinge upon an underwater linear array of corner reflectors, interactions among the individual corner reflectors take place, thereby giving rise to intricate acoustic scattering phenomena. Among these interactions, the mutual influence between two adjacent corner reflectors stands out as particularly significant. To delve deeply into the interaction patterns between two corner reflectors, we assembled an array by combining two eight-cell corner reflectors, designated as Corner Reflector I and Corner Reflector II, respectively. The specific layout is illustrated in Figure 6.

It is assumed that the incident acoustic wave is an ideal plane wave. The geometric center O of Corner Reflector I is designated as the origin of the coordinate system. Let θ represent the angle between the incident direction of the background acoustic field and the horizontal plane, and φ denote the angle between the incident direction of the background acoustic field and the *z*-axis. The deployment distance between Corner Reflector I and Corner Reflector II is D, and the deployment distance D is an integer multiple of the corner reflector’s edge length r. Each corner reflector has a side length r of 500 mm and a plate thickness h of 20 mm, and is fabricated from thin steel plates.

### 3.1. A Parallel Arrangement Where the Components Are Set at the Same Angle

Under the condition of a fixed size, the interaction pattern among corner reflectors is significantly influenced by their deployment spacing and deployment angles. Given the numerous possible deployment scenarios, in this manuscript, the deployment distance D between the corner reflectors is selected to be an integral multiple of the side length *r* of the corner reflectors. Moreover, the deployment posture of Corner Reflectors I and II is set as a parallel arrangement at the same angle, as shown in Figure 6.

**Figure 7:**
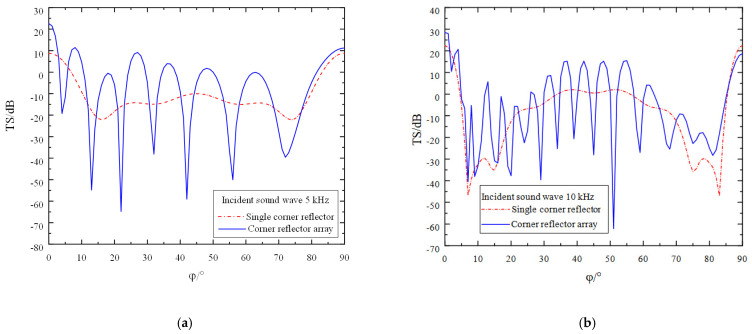

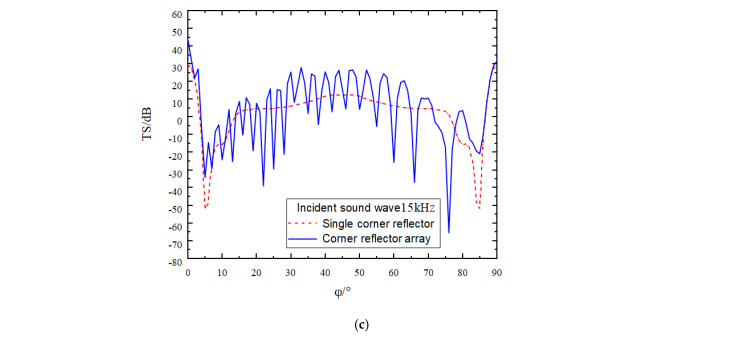
Target strength comparison of single corner reflector and two corner reflectors(D = 0 r). (**a**) The incident acoustic wave has a frequency of 5 kHz. (**b**) The incident acoustic wave has a frequency of 10 kHz. (**c**) The incident acoustic wave has a frequency of 15 kHz.

Due to the symmetric structure of the corner reflector linear array, the direction of the incident sound wave is set to *θ* = 90 and *φ* = 0° to 90°, with an amplitude of 1 Pa. The incident frequencies are set to 5 kHz, 10 kHz, and 15 kHz, respectively, and the deployment spacing D is 0 r. The simulation results are as shown in Figure 7.

An in-depth analysis of Figure 7 leads to the following conclusions:

(1) Owing to the interaction between two corner reflectors and the presence of a gap between them, the fluctuation characteristics of the target strength curve are significantly enhanced. The overall trend shows an up and down fluctuation around the target strength curve of the single corner reflector. As the incident frequency increases, the fluctuation of the target strength curve becomes more pronounced, while the amplitude of the fluctuation gradually decreases. At incident frequencies of 5 kHz, 10 kHz, and 15 kHz, the amplitudes of the fluctuations are approximately 60 dB, 40 dB, and 25 dB, respectively.
(2) As the number of corner reflectors increases, the relative cross-sectional area of the reflecting surface correspondingly expands, resulting in a significant increase in the target strength. By calculating the target strength at each sampling point, it is found that when the frequency of the incident acoustic wave is 5 kHz, the target strengths of the single corner reflector and the corner reflector array are −10.9 dB and −8.2 dB, respectively. At 10 kHz, the target strengths are −9.3 dB and −6.3 dB, respectively, and at 15 kHz, the target strengths are 2.1 dB and 4.4 dB, respectively.
(3) When the incident angle φ is changed, the variation trends of the target strengths of the corner reflector array and the single corner reflector are basically consistent. In the directions of 0° and 90°, due to specular reflection, the target strengths are relatively large, reaching 22 dB, 28 dB, and 40 dB, respectively. Around 5° and 85°, the target strengths are relatively small, being −54 dB, −40 dB, and −32 dB, respectively, indicating the existence of reflection blind spots. Even when the corner reflector array is deployed, the reflection performance in this angular range cannot be effectively improved.
(4) Since the linear array of corner reflectors contains multiple reflecting units, the incident acoustic wave causes each corner reflector to generate strong reflections. However, there is mutual influence among the corner reflectors, and complex scattering phenomena occur among them. Therefore, the scattered acoustic waves received at the field point are extremely complex. When the peaks and troughs of two acoustic waves meet, destructive interference occurs, causing the acoustic waves to cancel each other out. As a result, the scattered sound pressure at the field point decreases, leading to a reduction in the target strength. This is why, at certain incident angles, the target strength of the linear array of corner reflectors is lower than that of the single corner reflector.

**Figure 8:**
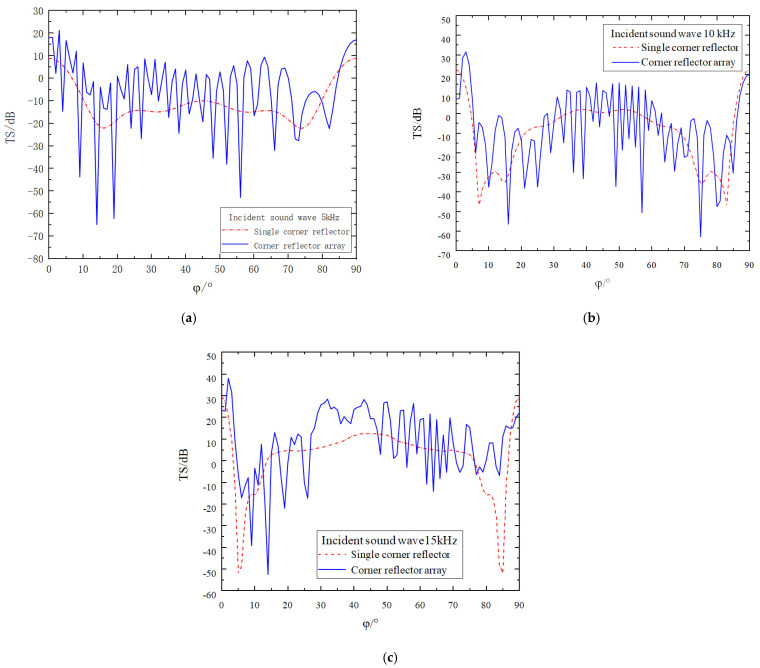
Comparison of target strength after increasing the spacing to 5 r. (**a**) The incident acoustic wave has a frequency of 5 kHz. (**b**) The incident acoustic wave has a frequency of 10 kHz. (**c**) The incident acoustic wave has a frequency of 15 kHz.

While keeping all the conditions required for the simulation calculation constant, the deployment spacing of the corner reflectors is increased to five times the side length *r* of these reflectors. The results obtained from the simulation operation are presented in Figure 8.

**Figure 9:**
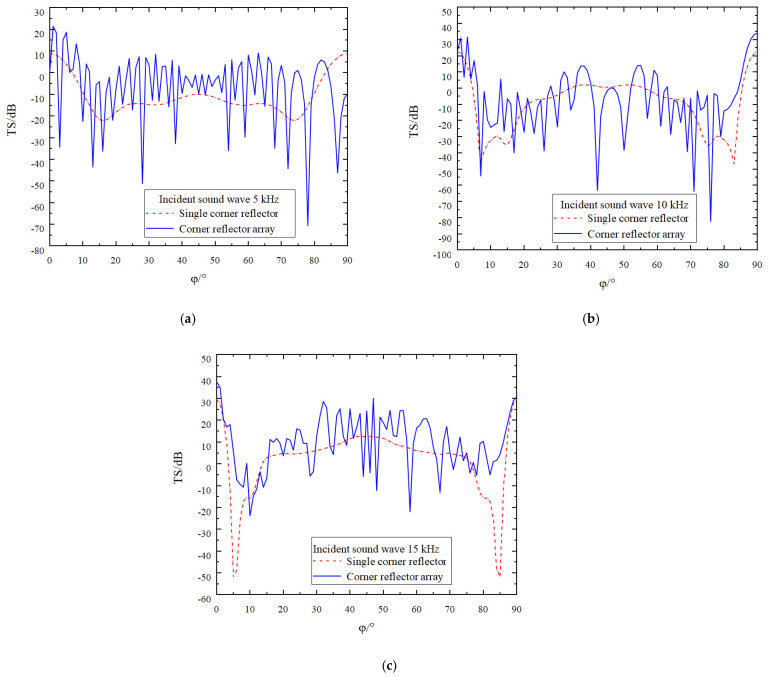
Comparison of target strength after increasing the spacing to 10 r. (**a**) The incident acoustic wave has a frequency of 5 kHz. (**b**) The incident acoustic wave has a frequency of 10 kHz. (**c**) The incident acoustic wave has a frequency of 15 kHz.

While keeping all the conditions required for the simulation calculation constant, the deployment spacing of the corner reflectors is increased to ten times the side length *r* of these reflectors. The results obtained from the simulation operation are presented in Figure 9.

As can clearly be observed from Figure 8 and Figure 9, there are numerous characteristics influencing the target strength of the corner reflectors. The specific conclusions are as follows:

(1) There are significant differences in target strength between the array composed of two corner reflectors and the single corner reflector. Generally speaking, the target strength of the two-corner reflector array is higher than that of the single corner reflector.
(2) As the deployment spacing between the corner reflectors increases, the degree of fluctuation in the target strength curve intensifies. Moreover, the amplitude of this fluctuation grows as the incident frequency rises. This is because an increase in frequency shortens the wavelength of the acoustic wave, resulting in a more concentrated distribution of acoustic waves within a unit space. This phenomenon is particularly prominent at incident frequencies of 5 kHz and 10 kHz.
(3) Despite the changes in the fluctuation of the target strength curve, its variation pattern remains largely stable, still fluctuating around the target strength curve of a single corner reflector.
(4) The target strength values of the corner reflectors generally remain constant. When the deployment distance is 5 r, by averaging the target strength values at each sampling point, the target strength values at the three incident frequencies are found to be −5.8 dB, −7.8 dB, and 8.7 dB, respectively. When the deployment distance is increased to 10 r, after averaging the target strength values at each sampling point, the target strength values at these three incident frequencies are −6.7 dB, −6.8 dB, and 9.3 dB, respectively.

### 3.2. The Corner Reflectors Are Precisely Configured with a Relative Rotation Angle of 45°

As can be learned from the simulation results in the previous section, when the single corner reflector is exposed to medium- and high-frequency acoustic waves, the target strength reaches a minimum value around φ = 5° and φ = 85°, indicating the presence of reflection blind spots. In practical engineering applications, this situation leads to poor decoying effects of the corner reflector.

The calculations in the previous section revealed that when two corner reflectors are arranged in parallel with the same angular orientation, reflection blind spots still exist around φ = 5° and φ = 85°. Evidently, simply increasing the number of corner reflectors cannot effectively compensate for this shortcoming.

Given that the target strength of the corner reflector reaches a maximum value around the incident angles of θ = 90° and φ = 45°, this implies that the corner reflector exhibits strong sound-reflecting capabilities within this angular range. Therefore, the overall acoustic scattering performance of the corner reflector array can be enhanced by adjusting the relative angle between the two corner reflectors.

**Figure 10:**
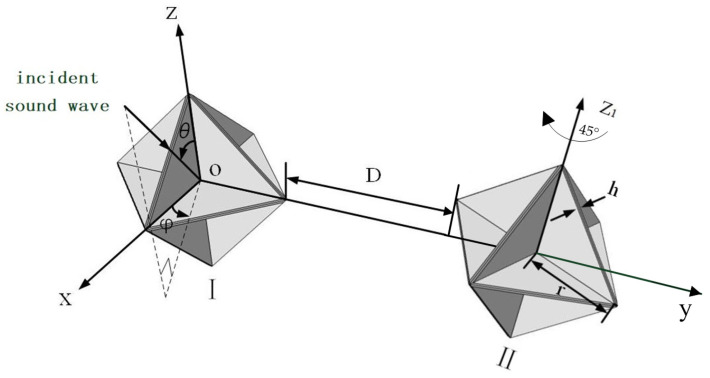
Corner Reflector II is rotated by 45° around the Z_1_ axis.

**Figure 11:**
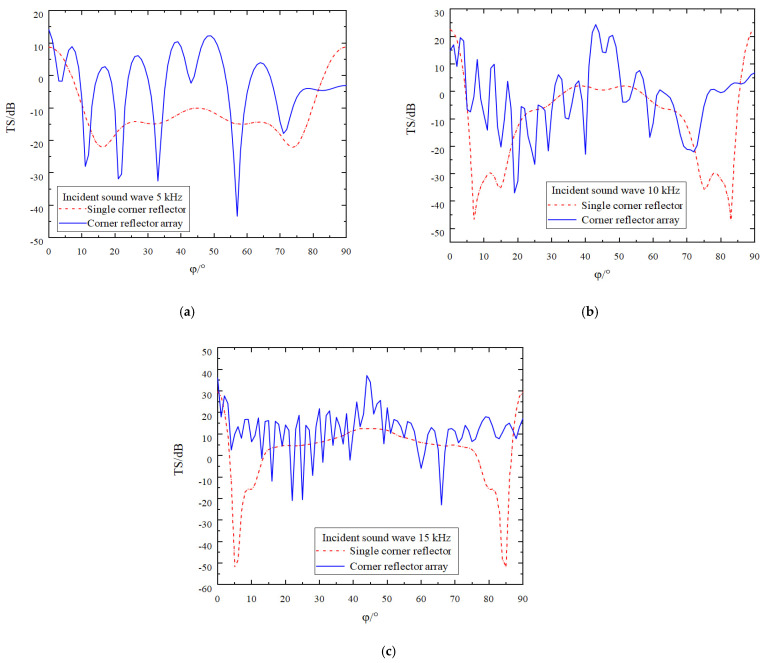
Target strength comparison of single corner reflector and two corner reflectors(D = 0 r. Relative Rotation Angle of 45°). (**a**) The incident acoustic wave has a frequency of 5 kHz. (**b**) The incident acoustic wave has a frequency of 10 kHz. (**c**) The incident acoustic wave has a frequency of 15 kHz.

As shown in Figure 10, the deployment posture has been adjusted. The position and angle of Corner Reflector I remain unchanged, while Corner Reflector II is rotated by 45° along the Z_1_ axis for deployment. Meanwhile, the deployment distance D between the two corner reflectors is continuously varied. The corresponding simulation results are presented in Figure 11.

The analysis of Figure 11 leads to the following conclusions:

(1) When the corner reflector array is deployed under the current angular conditions, as the incident angle φ changes, the target strength curve exhibits significant fluctuations due to the interaction among multiple corner reflectors. Moreover, the number of peaks and valleys in the target strength curve increases as the incident frequency of the acoustic wave rises.
(2) After calculating the target strength at each sampling point, it is found that when the incident acoustic-wave frequency is 5 kHz, the average target strength of the corner reflector array is −3.2 dB. At 10 kHz, the average target strength is −2.0 dB, and at 15 kHz, the target strength is 11.6 dB.
(3) Compared with the deployment angle used in the previous section, rotating one of the corner reflectors in the corner reflector array brings about two positive effects. Firstly, it effectively addresses the issue of low target strength around 5° and 85°. Secondly, it significantly enhances the overall target strength of the corner reflector array.

**Figure 12:**
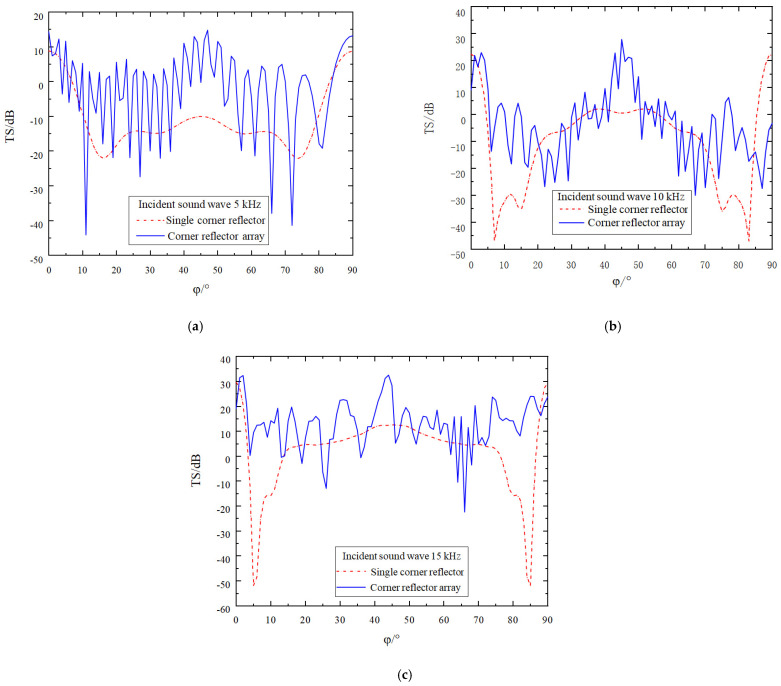
Comparison of target strength after increasing the spacing to 5 r (Relative Rotation Angle of 45°). (**a**) The incident acoustic wave has a frequency of 5 kHz. (**b**) The incident acoustic wave has a frequency of 10 kHz. (**c**) The incident acoustic wave has a frequency of 15 kHz.

While keeping all the conditions required for the simulation calculation constant, the deployment spacing of the corner reflectors is increased to five times the side length *r* of these reflectors. The results obtained from the simulation operation are presented in Figure 12.

**Figure 13:**
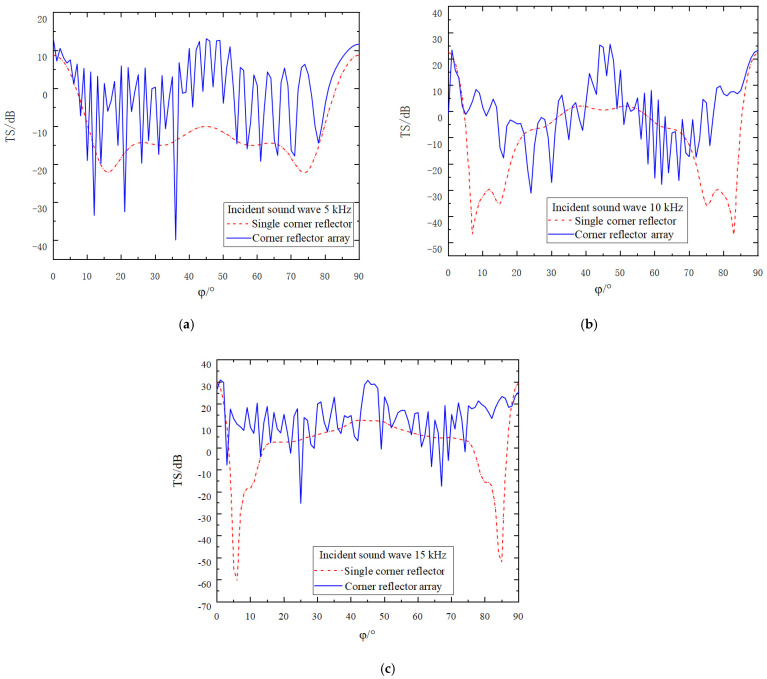
Comparison of target strength after increasing the spacing to 10 r (Relative Rotation Angle of 45°). (**a**) The incident acoustic wave has a frequency of 5 kHz. (**b**) The incident acoustic wave has a frequency of 10 kHz. (**c**) The incident acoustic wave has a frequency of 15 kHz.

While keeping all the conditions required for the simulation calculation constant, the deployment spacing of the corner reflectors is increased to ten times the side length *r* of these reflectors. The results obtained from the simulation operation are presented in Figure 13.

From Figure 12 and Figure 13, we can clearly observe the following phenomena, and the relevant conclusions are as follows:

(1) As the deployment spacing between the corner reflectors increases, the gaps between them also widen, resulting in a more frequent occurrence of peaks and valleys in the target–strength curve. Moreover, the density of these peaks and valleys in the target strength curve further increases as the incident frequency rises.
(2) Comparing the two deployment methods—namely the parallel deployment of two corner reflectors at the same angle and deployment with a relative rotation of 45°—at an incident frequency of 5 kHz, due to the relatively long wavelength of the acoustic wave, there is no significant difference in the improvement of the target strength between the two deployment methods. However, when the incident frequency reaches 10 kHz and 15 kHz, thanks to the reinforcement effect of Corner Reflector II, within the angular range around 5° and 85°, the deployment method with a relative rotation of 45° can increase the target strength by approximately 20–30 dB. This significantly reduces the reflection blind spots of the two-corner reflector array.
(3) The target strength values of the corner reflectors generally remain relatively stable. When the deployment distance is 5r, by averaging the target strength values at each sampling point, the target strength values at the three incident frequencies are −2.2 dB, −3.5 dB, and 13.0 dB, respectively. When the deployment distance is 10r, after averaging the target strength values at each sampling point, the TS values at these three incident frequencies are −2.6 dB, −3.8 dB, and 12.6 dB, respectively.

## 4. Validation by Means of Pool Experiments

To verify the accuracy of the simulation data calculation and analysis, it is necessary to conduct a pool verification experiment.

This experiment was carried out in a reverberation pool. After formulating and adopting a reasonable experimental scheme, two underwater eight-cell triangular corner reflectors and corresponding equipment deployment devices were carefully designed and fabricated. Subsequently, a test was conducted on the acoustic scattering characteristics of the array composed of these two corner reflectors, and the test results were carefully compared with the simulation calculation results.

### 4.1. Systematic Design and Production of the Corner Reflector Model Along with the Corresponding Hoisting Device

To effectively verify the simulation results of the acoustic scattering characteristics of the corner reflectors and the linear arrays composed of them, we meticulously fabricated two eight-cell triangular corner reflectors equipped with air cavities, along with a corresponding deployment device.

The side length of each corner reflector was set to 250 mm, the thickness of the air cavity was 16 mm, and the thickness of the metal sheets on both sides was 2 mm. To prevent the corner reflectors from deforming under water pressure, a certain number of reinforcing ribs were reasonably installed inside them, significantly enhancing their pressure resistance capacity. Additionally, bolts and nuts were provided on the corner reflectors to ensure that they could be firmly fixed to the deployment device and could also be flexibly rotated at various angles.

**Figure 14:**
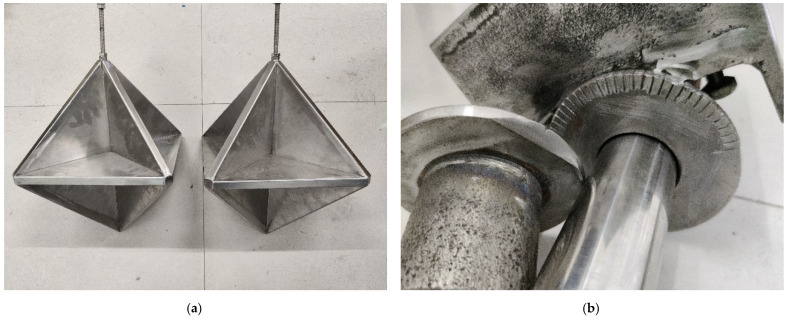
Corner reflector physical model and rotary placement device. (**a**) Physical corner reflector. (**b**) Scale-adjustable disc device.

The deployment device was carefully designed with screw holes at specific intervals, enabling the linear array of corner reflectors to easily adjust the deployment distance. To ensure that the corner reflectors could be rotated at precise angles, a graduated disc was specially designed. The specific structures of the corner reflectors and the deployment device are shown in Figure 14.

### 4.2. Comprehensive Design for the Pool-Based Experiment

**Figure 15:**
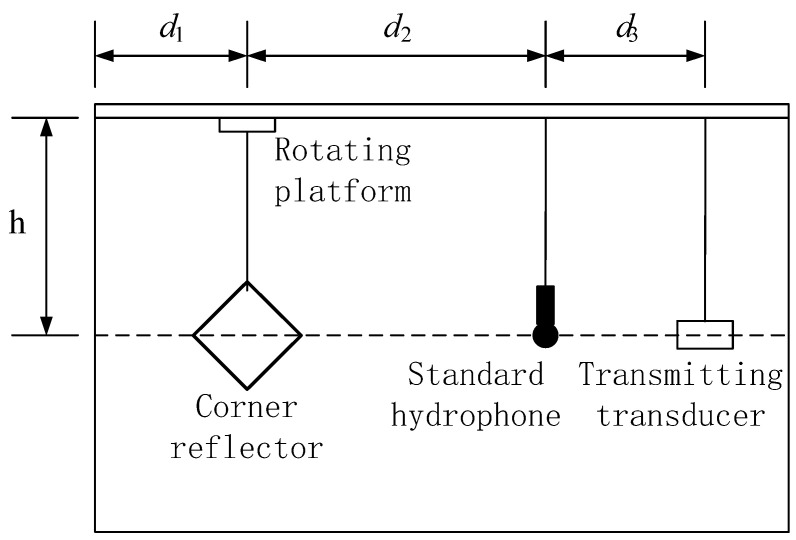
Schematic diagram of experimental layout.

To effectively meet the experimental requirements, a pool test was carried out in a reverberation pool. The pool at the test site has a volume specification of 6 m × 4 m × 4 m. Above the pool, sliding rails and fixing devices are installed, which fully met the requirements for the deployment and positioning of the transmitting transducer, hydrophone, and underwater corner reflectors; the specific deployment is shown in Figure 15.

**Figure 16:**
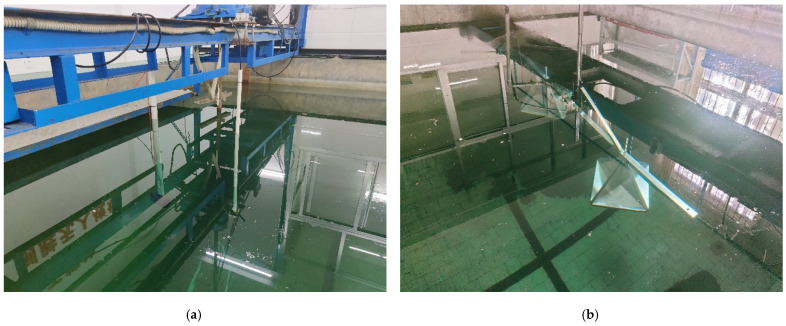
Site of experimental layout. (**a**) The detailed and actual situation of the deployment for the transmitting transducer and the hydrophone. (**b**) The detailed real-time status of the corner reflector deployment, showing every aspect of its placement.

As can clearly be observed from Figure 15, to ensure the accuracy and reliability of the experiment, different yet suitable deployment methods were adopted for the three main experimental devices. Specifically, the transmitting transducer and the corner reflector were fixed in position using a rigid structure. This rigid fixation method could effectively resist external interference and ensured the stability of the devices’ positions. Meanwhile, the hydrophone was suspended by a flexible rope. The flexible rope could not only flexibly adjust the position of the hydrophone, but also buffered the impact of external vibrations to a certain extent. Through such a carefully arranged deployment, the geometric centers of the three devices were precisely aligned on the same horizontal line. The actual on-site situation of the experimental deployment is shown in Figure 16, which intuitively presents the practical implementation of the entire deployment plan.

The equipment used in this experiment included several key components, specifically a pulse signal generator, a KEMOV BF40 filter amplifier, a transmitting transducer, an RHSA-30 hydrophone, a data acquisition device, and a power supply. These devices work together to complete the tasks of signal generation, transmission, reception, and acquisition.

In this experiment, the corner reflector has an edge length of 0.25 m. The parameters are set as follows: d_1_ = 2 m, d_2_ = 3 m, d_3_ = 0.5 m, and h = 1.5 m. According to the formula *L*^2^/*λ*, under the condition of an incident sound source at 15 kHz, the distance from the corner reflector to the standard hydrophone d_2_ = 3 m is selected to satisfy the far-field condition. The incident sound wave is a CW (continuous wave) pulse signal with a pulse period of 2 s and a pulse width of 1 millisecond at a frequency of 15 kHz. By using a relatively narrow pulse width and ensuring no less than ten waves within a single pulse period, stable scattering target intensity is achieved, minimizing the impact of the reverberation waves from the incident pulse signal on the target reflection waves. Under the premise of an unchanging acoustic center, the corner reflector is rotated, and data are collected every 3 degrees. The influence of angular variation on the target strength value of the corner reflector is measured. The hydrophone is connected to an oscilloscope, on which the changes in target intensity echo waves are observed and recorded.

The reverberation waves from the pool walls, bottom, and surface are collectively referred to as reverberation waves. The signal received by the standard hydrophone in the reverberation pool consists of direct waves from the transmitter, reverberation waves, and scattered waves from the corner reflector. Among them, the reverberation waves are further divided into surface reflection waves, bottom reflection waves, side-wall reflection waves, and waves reflected by the transmitter against the pool wall. By reasonably placing the transmitter, standard hydrophone, and corner reflector, the time for the hydrophone to receive the direct sound wave from the transmitter is 0.33 ms, the side-wall reflection wave is 1.3 ms, the surface reflection wave is 1 ms, the bottom reflection wave is 1.67 ms, and the wave reflected by the hydrophone against the pool wall arrives at 6 ms, while the echo from the corner reflector target arrives at 4.3 ms. By setting the experimental equipment layout to minimize the overlap and interference of different sound waves arriving at the hydrophone, it is beneficial to observe the acoustic scattering signal of the underwater corner reflector on the oscilloscope.

The hydrophone converts acoustic signals into electrical signals, which are then displayed as voltage values on an oscilloscope. The formula for calculating the target strength value is based on

(14) $$TS=20\mathrm{lg}\frac{U_{b}}{U_{d}}+20\mathrm{lg}\frac{d_{2}}{d_{3}}+20\mathrm{lg}(d_{3}+d_{2})$$

In the equation, *d*_3_ is the distance between the transmitter and the standard hydrophone, *d*_2_ is the distance from the hydrophone to the target corner reflector, *U_b_* represents the voltage value of the corner reflector’s echo signal, and *U_d_* denotes the voltage value of the direct wave. The values of *d*_1_, *d*_2_, and *d*_3_ are established in Figure 15, and *U_b_* and *U_d_* are determined by reading the oscilloscope.

### 4.3. Experimental Data Collection and In-Depth Analysis

To gain a deeper understanding of the acoustic characteristics of a linear array composed of two corner reflectors underwater, we meticulously carried out an underwater experiment. The incident angle of the acoustic wave is clearly and intuitively presented in Figure 6. During the experiment, the two corner reflectors were arranged at the same deployment angle, and the spacing between them was precisely set at 1000 mm to ensure the accuracy and repeatability of the experimental conditions.

**Figure 17:**
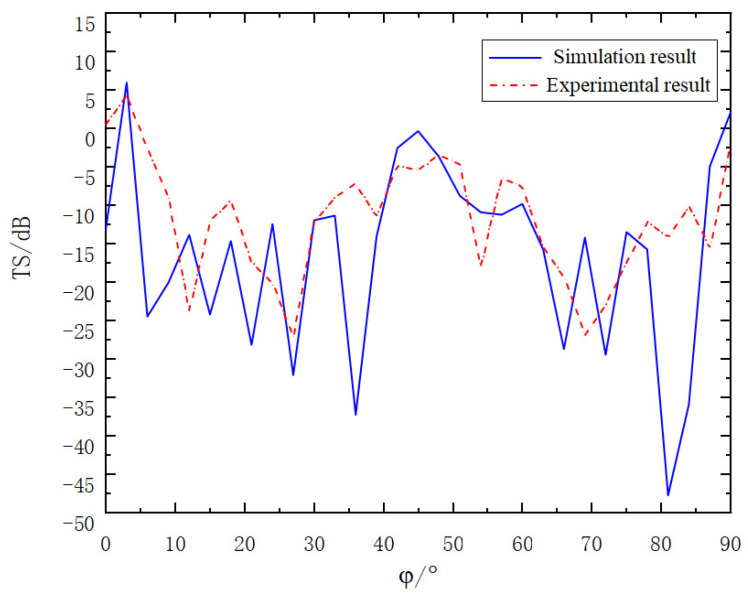
Experimental results of corner reflector array.

After the experiment, we systematically and meticulously sorted out a large amount of collected experimental data. To verify the reliability of the experimental results and evaluate the accuracy of the simulation model, we comprehensively compared the sorted experimental data with the pre-calculated simulation results. The comparison results are clearly shown in Figure 17, which provides an important basis for our further analysis of the differences and connections between the experiment and the simulation.

As can clearly be observed from the figure, when the two-corner reflector array is deployed at the same angle, the acoustic characteristics follow a specific pattern. At an incident angle φ = 3°, the target strength reaches its maximum value. Specifically, the simulation result shows this maximum to be 6.25 dB, while the experimental result is 4.98 dB. Additionally, there is another relative maximum when the incident angle φ is close to 45°. At this point, the target strength obtained from the simulation is 0.21 dB, and the value measured in the experiment is −4.36 dB.

Upon further analysis of the entire range of incident angles from φ = 0° to φ = 90°, it is evident that the simulation results and the experimental results are in general agreement in terms of the overall trend. This indicates that under the experimental conditions, the adopted simulation model can accurately predict the acoustic response of the two-corner reflector array, providing strong support for relevant theoretical research and practical applications.

**Figure 18:**
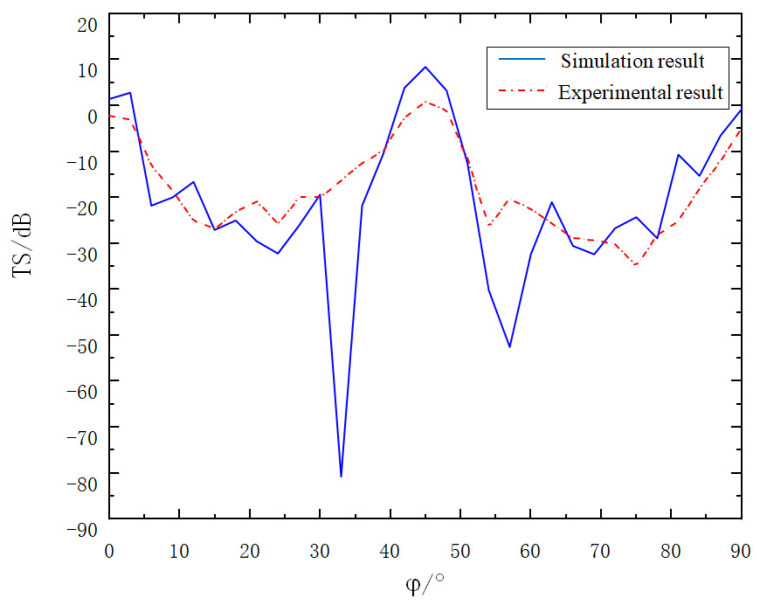
Experimental results of corner reflector array after changing the arrangement angle.

To further explore the acoustic scattering characteristics of the corner reflector linear array after changing the deployment angles, the deployment angle of Corner Reflector I was set to remain unchanged, while Corner Reflector II was rotated by 45° along the Z_1_-axis. With other conditions kept the same, a comparison between the experimental data and the simulation results is presented in Figure 18.

As can be seen from Figure 18, after the two corner reflectors are deployed with a relative rotation of 45°, the fluctuation of the target strength curve decreases and the value increases. The maximum value of the target strength is obtained when the incident angle φ = 45°. The simulation result and the experimental result are 9.78 dB and 1.02 dB respectively, and the two results are in good agreement.

As can clearly be observed from Figure 17 and Figure 18, the variation trends of the target strength curves presented by the experimental results and the simulation results are basically the same. Upon meticulous analysis of the data, the following can be concluded:

(a) Compared with the single corner reflector, the corner reflector linear array can significantly enhance the overall target strength to a considerable extent. This indicates that the size of the target reflection area has a great influence on the target strength value.
(b) The corner reflector linear array can also effectively optimize the problem of the low target strength of the single corner reflector at around 20° and 70°. This optimization effect is more prominent when the corner reflectors are deployed with relative rotation. The reason is that when one corner reflector is at the blind angle, this is exactly the angular range where the reflection intensity of the other corner reflector is the largest.
(c) Based on the above-mentioned advantages, in practical engineering applications, the deployment method of the relative rotation of corner reflectors can be considered to ensure that the target strength values in all directions meet the requirements of the target simulation.

### 4.4. Comprehensive Error Analysis

From the experimental results, it can be observed that although there is a certain deviation between the results calculated by the COMSOL simulation software (COMSOL 5.4) and the measured results, the variation trends of the TS curves are basically consistent, and the variation laws of the target strength at various angles are generally the same. However, there are still some errors between the simulation calculation data and the experimental measurement data.

After analysis, the main reasons for these errors are as follows:

(1) The processing technology of the eight-cell corner reflector is relatively complex. There are manufacturing errors in the perpendicularity of the flat plates of each reflecting surface. This causes the angle of the acoustic wave reflection to change, and the wave fails to return to the field point.
(2) The reflecting surface plates of the corner reflector are composed of “thin steel plate–air–thin steel plate”. The reinforcing ribs, welded to enhance pressure resistance, affect the scattering of acoustic waves. The acoustic waves will undergo multiple refractions in the reinforcing ribs, resulting in a certain amount of acoustic wave loss.
(3) The reinforcing ribs cannot completely prevent the corner reflector from deforming under water pressure. Therefore, during underwater tests, the flat plates of the corner reflector will be depressed inward due to water pressure, causing a deviation in the reflection direction of the reflected wave.
(4) The acoustic centers of the transmitting transducer, hydrophone, and corner reflector deployed in the experiment are not in a straight line, resulting in alignment errors. As a result, the received signal cannot be guaranteed to come from the geometric center of the target.
(5) Due to site limitations, for non-anechoic pools, there are interfering factors such as pool-wall reverberation.

## 5. Conclusions

The influence of changing the deployment distance and angle of each unit in the linear array on the acoustic scattering characteristics of the linear array was analyzed, and a method to improve the reflection blind areas of corner reflectors was proposed. The specific conclusions are as follows:

(1) With the increase in the number of corner reflectors in the linear array, the overall target strength is significantly enhanced. The target strength of the two-corner reflector array is approximately 5 dB greater than that of the single corner reflector, mainly due to the superposition contribution of the scattered acoustic waves of multiple reflecting units at the field point.
(2) The scattered acoustic waves of the metal-plate corner reflectors in the linear array will interact with each other, causing the target strength curve to fluctuate violently. The number of fluctuation peaks and valleys increases with the increase in the number of corner reflectors.
(3) The fluctuation amplitude of the target strength curve decreases with the increase in incident frequency. The fluctuation amplitude is the largest when the incident frequency is 5 kHz and the smallest when it is 15 kHz.
(4) By reasonably setting the deployment angles of each corner reflector and complementing the angles with strong reflection ability and those with weak reflection ability, the problems of low target strength and the existence of reflection blind areas of corner reflectors at around 5° and 85° can be optimized.

## Data Availability

The data presented in this study are available upon request from the corresponding author.

## References

[1] Liao W.J., Hou Y.C., Tsai C.C., Hsieh T.H., Hsieh H.J. (2018). Radar Cross Section Enhancing Structures for Automotive Radars. IEEE Antennas Wirel. Propag. Lett..

[2] Ratni B., de Lustrac A., Piau G.-P., Burokur S.N. (2018). Active metasurface for reconfigurable reflectors. Appl. Phys. A.

[3] Zhu Y.G., Ai X., Wang W.D. (2023). Design method of new reconfigurable radar corner reflector with multiple electromagnetic characteristics. Chin. J. Radio Sci..

[4] Gan L., Sun G., Feng D., Li J. (2022). Characteristics of an Eight-Quadrant Corner Reflector Involving a Reconfigurable Active Metasurface. Sensors.

[5] Ferrer P.J., Lopez-Martinez C., Aguasca A., Pipia L., Gonzalez-Arbesu J.M., Fabregas X., Romeu J. (2011). Transpolarizing Trihedral Corner Reflector Characterization Using a GB-SAR System. IEEE Geosci. Remote Sens. Lett..

[6] Hu H., Zhang L., Zhang X. (2018). A study on equipment developments and operational using of ship born gas-filled anti-missile multi-cornerreflector. Def. Technol. Rev..

[7] Zhang Z., Zhao Y. (2018). Analysis of electromagnetic scattering characteristic for new type icosahedrons triangular trihedral corner reflectors. Command Control Simul..

[8] Li H., Chen S., Wang X. (2022). Study on characterization of sea corner reflectors in polarimetric rotation domain. Syst. Eng. Electron..

[9] Zhao F., Qiu M.Q., Ai X.F., Xu Z.M. (2023). Comparative Analysis of Monostatic and Bistatic RCS Characteristics for Typical Corner Reflectors. Mod. Def. Technol..

[10] Ke X. (2025). Angular Reflector Return Intensity Characteristics. Partially Coherent Optical Transmission Theory in Optical Wireless Communication. Optical Wireless Communication Theory and Technology.

[11] Xia L., Wang F., Pang C., Li N., Peng R., Song Z., Li Y. (2024). An Identification Method of Corner Reflector Array Based on Mismatched Filter through Changing the Frequency Modulation Slope. Remote Sens..

[12] Guo T. (2019). RCS Computation of Towed Passive False Target. Master’s Thesis.

[13] Chen W.J. (2012). Research on Underwater Acoustic Corner Reflector Properties. Ph.D. Thesis.

[14] Liang J.J., Yu Y., Chen W.J., Hu S.W., Wang H., Wang Y.Z. (2017). A modified shooting and bouncing beams method for fast calculating the acoustic scattering field of circular trihedral corner reflector. Tech. Acoust..

[15] Chen W.J., Sun H. (2015). Study on the acoustic backscattering characteristics of underwater corner reflector. J. Inf. Comput. Sci..

[16] Avdeev I.S. (2010). The use of the boundary element method in solving the problems of sound scattering by an elastic noncircular cylinder. Acoust. Phys..

[17] Lin L., Lv J., Li S. (2024). An adaptive finite element DtN method for the acoustic-elastic interaction problem. Adv. Comput. Math..

[18] Zhao X.F., Cui H.C., Dong R. (2019). Simulation Experiments of Lamb Wave’s Scattering Sound Field on Underwater Elastic Plate. Digit. Ocean Underw. Warf..

[19] Luo Y., Chen X. (2019). Acoustic Scattering Characteristics of Underwater Air-filled Cavity Corner Reflector. Acta Armamentarii.

[20] Luo Y., Wang J.Y., Xie T.T. (2020). A Foam Interlayer Method for Improving on the Acoustic Reflection Capability of Underwater Corner Reflector. Acta Armamentarii.

[21] Xie T.T., Luo Y., Xiao D.W. (2022). Analysis of Acoustic Scattering Characteristics of Underwater Non Orthogonal Acoustic Corner Reflectors. J. Underw. Unmanned Syst..

[22] Wang X.F., Meng H.R., Yu F.X. Research on Information Modeling Technology of Underwater Large Structures Based on FEM and BEM. Proceedings of the 18th Symposium on Underwater Noise of Ships Dalian Institute of Measurement and Control Technology.

[23] Hu H.W., Wang Z.W., Xu Y.M., Chen L.L. (2022). A study on the isogeometric finite element-boundary element method for the stochastic analysis of structural acoustic coupling. J. Vib. Shock.

[24] Feng J.L., Wang H.H., Zhao Y., Wang Y.N., Du L.G. (2018). Finite Element-Boundary Element Method Simulation for Acoustic-Vibration Problems of Spacecraft Structures. Equip. Environ. Eng..

